# Expression genome-wide association study identifies key regulatory variants enriched with metabolic and immune functions in four porcine tissues

**DOI:** 10.1186/s12864-024-10583-w

**Published:** 2024-07-11

**Authors:** Samin Farhangi, Marta Gòdia, Martijn F.L. Derks, Barbara Harlizius, Bert Dibbits, Rayner González-Prendes, Richard P.M.A. Crooijmans, Ole Madsen, Martien A.M. Groenen

**Affiliations:** 1 Animal Breeding and Genomics, Wageningen University and Research, Wageningen, The Netherlands; 2 Topigs Norsvin Research Center, ’s-Hertogenbosch, The Netherlands; 3 Ausnutria BV, Zwolle, The Netherlands

**Keywords:** eGWAS, RNA-seq, Pig, Transcription factors, Regulatory variants

## Abstract

**Background:**

Integration of high throughput DNA genotyping and RNA-sequencing data enables the discovery of genomic regions that regulate gene expression, known as expression quantitative trait loci (eQTL). In pigs, efforts to date have been mainly focused on purebred lines for traits with commercial relevance as such growth and meat quality. However, little is known on genetic variants and mechanisms associated with the robustness of an animal, thus its overall health status. Here, the liver, lung, spleen, and muscle transcriptomes of 100 three-way crossbred female finishers were studied, with the aim of identifying novel eQTL regulatory regions and transcription factors (TFs) associated with regulation of porcine metabolism and health-related traits.

**Results:**

An expression genome-wide association study with 535,896 genotypes and the expression of 12,680 genes in liver, 13,310 genes in lung, 12,650 genes in spleen, and 12,595 genes in muscle resulted in 4,293, 10,630, 4,533, and 6,871 eQTL regions for each of these tissues, respectively. Although only a small fraction of the eQTLs were annotated as *cis*-eQTLs, these presented a higher number of polymorphisms per region and significantly stronger associations with their target gene compared to *trans*-eQTLs. Between 20 and 115 eQTL hotspots were identified across the four tissues. Interestingly, these were all enriched for immune-related biological processes. In spleen, two TFs were identified: *ERF* and *ZNF45*, with key roles in regulation of gene expression.

**Conclusions:**

This study provides a comprehensive analysis with more than 26,000 eQTL regions identified that are now publicly available. The genomic regions and their variants were mostly associated with tissue-specific regulatory roles. However, some shared regions provide new insights into the complex regulation of genes and their interactions that are involved with important traits related to metabolism and immunity.

**Supplementary Information:**

The online version contains supplementary material available at 10.1186/s12864-024-10583-w.

## Background

In the last two decades, genome-wide association studies (GWAS) have facilitated the discovery of genetic variants associated with traits and diseases in livestock species [1]. Most of these variants are located within non-coding genomic regions and are enriched for gene regulatory elements [2]. Among all genetic variants, Single Nucleotide Polymorphisms (SNPs) have been extensively used to study their contribution to phenotype variability by regulating gene activity. SNPs associated with gene expression levels are known as expression quantitative trait loci (eQTLs) and are commonly identified through expression GWAS (eGWAS). Compared to GWAS, eQTL mapping can be more powerful in detecting statistically significant genetic effects and revealing inherent biological meaning for complex polygenic traits [3], which in breeding programs enables the identification of new selection targets [4].

Efforts in porcine eQTL mapping have been mostly focused on commercial relevant phenotypes such as growth, body composition, carcass, meat quality and reproduction. Those studies are mostly targeting a single tissue type including, for example, muscle [5–10], colon [11], liver [8, 12], adipose tissue [13, 14], and sperm [15]. Nevertheless, knowledge on genetic variation in other relevant organs such as spleen or lung with roles associated to disease resistance and animal robustness remains to be elucidated [16].

Understanding genome regulation to achieve a complete picture of genotype-phenotype interaction has become a major focus of interest for the livestock scientific community [17]. For this, efforts from recent projects [18] as well as the Farm animal Genotype-Tissue Expression (FarmGTEx) consortium have resulted in a comprehensive public resource of genetic regulatory effects across tissues and cells in pigs (PigGTEx) [19]. Using over 9,000 public transcriptome datasets obtained from different tissue and cell types, sex and porcine breeds, hundreds of key regulatory elements and genomic regions of interest have been pin-pointed in the pig genome [19]. This large resource also showed that genotype and transcriptome of matching individuals remain scarce because gene expression changes with age, environment, sex, genetic background and experimental design, as well as that imputed variants from a limited number of animals can produce considerable noise [19].

Our study aims to provide additional resources to the community to understand pig genome regulation in tissues relevant to production as well as for their involvement in health and immunity. To achieve this, a total of 100 female three-way crossbred pigs were analyzed to discover the association between genetic variants and changes of gene expression levels in four different tissues: liver, lung, spleen, and muscle. We identify novel putative regulatory regions and eQTL hotspot regions that show tissue-specific patterns as well as common regulatory polymorphisms. We also investigate putative regulatory pathways based on transcription factors (TFs) related to *trans*-eQTL hotspots.

## Methods

### Sample collection, RNA extraction and sequencing

A total of 100 gilts from a three-way cross breed ((Landrace*Large White)*Synthetic boar line) from Topigs Norsvin were randomly selected from the study Luo et al., [20]. Piglets were raised on a conventional commercial farm and kept with the sows in farrowing pens until weaning at on average 4 weeks of age. Then, all piglets were housed in same sized pens (1.2 × 2.85 m^2^) and fed with a standard commercial diet for growing pigs *ad libitum* [20]. Litter ID for family relatedness is included in Additional file 1. The animals were slaughtered at two months old (7 to 9 weeks old) and were divided into two batches of 53 and 47 samples, based on slaughter day. Tissue samples from liver, lung, spleen, and skeletal muscle from each animal were collected immediately after slaughter and stored in RNAlater (Thermo Fisher Scientific) at -80 °C until further use.

Total RNA was extracted from the 400 samples (100 samples x 4 tissues). For lung, liver, and spleen the QIAshredder homogenizer kit (Qiagen) was used, followed by the RNeasy kit (Qiagen) manufacture’s guidelines. For muscle tissue, a preceding Qiazol (Qiagen)-chloroform RNA extraction method [21] was employed to improve the RNA amounts outcome, and followed by the RNeasy kit (Qiagen). Extracted RNA was then subjected to quality control parameters including quantification with NanoDrop 1000 (Thermo Fisher) and Qubit RNA BR Assay kit (Invitrogen), and their integrity validated with Bioanalyzer Agilent RNA 6000 Nano kit (Agilent Technologies). RNA isolation was successful for all samples, obtaining an average of 63.6 ng/µl for liver (min 23.2 ng/µl; max 98.8 ng/µl), 63.6 ng/µl for lung (min 34.2 ng/µl; max 112.0 ng/µl), 71.1 ng/µl for spleen (min 38.8 ng/µl; max 120.0 ng/µl) and 68.9 ng/µl for muscle (min 26.0 ng/µl; max 114.0 ng/µl). All samples presented RNA Integrity (RIN) values ≥ 7.

RNA was poly(A) enriched using NEBNext^®^ Poly(A) mRNA Magnetic Isolation Module (New England Biolabs) and used for library preparation with the NEBNext^®^ Ultra™ Directional RNA Library Prep Kit (New England Biolabs). Samples were sequenced as stranded 150 bp paired end reads in an Illumina NovaSeq 6000 sequencing platform.

### Gene annotation and quantification

The bioinformatics pipeline of the study is summarized in Additional Fig. 1. RNA-seq reads were evaluated for quality control with the FastQC 0.11.9 software (https://www.bioinformatics.babraham.ac.uk/projects/fastqc/). Low-quality reads and adaptors were trimmed with Trim Galore 0.3.7 (https://www.bioinformatics.babraham.ac.uk/projects/trim_galore/) using default parameters. Filtered reads were then aligned to the Scrofa11.1 genome [22] using STAR 2.7.8 [23] with default parameters. Reads with an alignment MAPQ score less than 30 were removed using SAMtools 1.1.19 [24]. Genes annotated in the porcine genome (Ensembl 104) were quantified using htseq-count 0.11. 1 [25] with “--stranded = reverse”, followed by TMM normalization with EdgeR [26]. Genes with a TMM-normalized count per million (CPM) equal to or above one, were considered expressed and used for further analysis. Quality control of expression data was conducted to assess overall variability, including batch effects evidenced by the clustering of samples. This assessment was performed with a Principal Component Analysis (PCA) across all samples and tissue types using the R package DESeq2 [27] (Additional Fig. 2).

### DNA extraction, genotyping, and data filtering

DNA was extracted from spleen using a phenol-chloroform based method [28]. Quality and quantity of the DNA were assessed with NanoDrop 1000 (Thermo Fisher) and Qubit DNA BR Assay kit (Invitrogen). DNA fragment integrity was validated using agarose gel electrophoresis (1.5%, 120 V and 30 min). DNA was genotyped with the high-density (660 K markers) Axiom™ Porcine Genotyping Array (Thermo Fisher Scientific). The resulting genotyping dataset was stringently filtered using PLINK 1.9 [29] by excluding SNPs that had a minor allele frequency lower than 0.01, strong deviation from Hardy–Weinberg equilibrium (*P*-value ≤ 1e-12), more than 10% missing genotypes, and SNPs located on the Y chromosome, scaffolds or unmapped positions. The SNP positions were updated from Sscrofa 10.2 to the Sscrofa11.1 reference genome assembly [22] using PLINK 1.9 [29].

As a quality control step, genotypes of the crossbred animals included in this study were compared with their parental purebred lines (Large White, Landrace, and Synthetic boar line) [30]. PCA analysis was conducted in PLINK 1.9 [29] using the dimension reduction “--pca” option. Visualization was performed with the R package “ggplot2” [31].

### eGWAS study

eGWAS included all 100 samples with filtered genotypes and normalized expression data using a mixed model. This was performed independently for each of the four tissues. Only genes with average expression ≥ 1 CPM across samples were included. Single-SNP association analysis was performed with GCTA 1.25 [32] with the following model:

$${Y}_{ijk}=\mu +{Batch}_{j}+ \delta {SNP}_{i}+{u}_{k}+{e}_{ijk}$$

where ($${Y}_{i})$$ is the CPM gene expression modeled as a function of the population mean (µ), correcting for the fixed effect batch ($${Batch}_{j}$$) based on slaughter day; *δ* is additive effect of each candidate SNP to be tested for association, estimated as a regression coefficient on the corresponding (values 0, 1, 2) of the $$i$$*SNP*; $${\text{u}}_{k}$$ is the polygenic effect, that follow a normal distribution with $$\text{u}\sim N\left(0,G{\sigma }_{u}^{2}\right)$$, where G is the genomic relationship matrix calculated using the filtered SNPs and based on the methodology of [32], and $${\sigma }_{u}^{2}$$ is the additive genetic variance; and $${e}_{ijk}$$is the residual term.

The association between a SNP and gene expression was declared significant when Benjamini-Hochberg adjustment (*q*-value) ≤ 0.05 [33]. Significantly associated SNPs with consecutive distances shorter than 10 Mb were considered to belong to the same eQTL interval [12]. To reduce false positives calls, only eQTL with intervals ≥ 3 significant SNPs were considered. Linkage disequilibrium (LD) across SNPs within the same eQTL interval was evaluated for a set of 5 random eQTL regions per tissue. The analysis was conducted with PLINK 1.9 [29] with default settings.

### cis and trans-eQTLs

The SNPs identified were classified based on their genomic location. We assessed the distance between the most significant SNP of the eQTL and the transcriptional start position of its associated gene. Then, they were categorized into three groups: (i) *cis*-eQTL: the most significant eQTL SNP and its associated gene were ≤ 1 Mb distance; (ii) *trans*-eQTL-I: the SNP and its associated gene were located > 1 Mb distance on the same chromosome; (iii) *trans*-eQTL-II: the SNP and its associated gene were located on different chromosomes.

The variation in significance (*q*-value) for the distance between eQTL and its associated gene was analyzed using a one-way analysis of variation (ANOVA) and subjected to multiple comparison tests with Fisher’s Least Significant Difference (LSD) (*P*-value < 0.05) using R 1.1.10 [34] .

### Gene ontology and pathway analysis and overlap with transcription factors

The software ShinyGO 0.77 [35] was used for Gene Ontology (GO) and KEGG pathway enrichment analyses with default parameters, providing genes (CPM ≥ 1) as background for each tissue respectively and *S.scrofa* as background species. Only GO terms and KEGG pathways with a False Discovery Rate (FDR) ≤ 0.05 were considered significant. Candidate hotspot eQTL associated genes were queried against transcription factors (TFs) and cofactors (TcoFs) using the AnimalTFDB 4.0 [3] database.

The putative regulatory role of *trans*-eQTL hotspots acting as TFs was also investigated. For this, DNA motifs were first extracted from the hotspot eQTL genes’ promoters. The promoter sequence 900 bp upstream from the Transcription start site (TSS) of each gene [36] was extracted with BioMart [37] web tool. Sequences were then submitted to MEME Suite [38] web tool, with default parameters but with a maximum number of 10 motifs. Then, using Tomtom tool [39], DNA sequences were queried to both JASPAR [40] and AnimalTFDB 4.0 [3] TF and TcoFs databases for matching hits to minimize database limitations and their potential false positives or negatives queries. Only those hits with *q*-value ≤ 0.05 were considered significant.

## Results

### RNA-seq and bioinformatics analyses

The RNA-seq resulted in an average of 45.0, 42.3, 44.1 and 44.6 Million reads per sample in liver, lung, spleen, and muscle, respectively, and 99.9% of them passed quality control filters. On average, 95.1%, 93.8%, 92.0% and 94.7% of the reads mapped to the porcine genome in liver, lung, spleen, and muscle, respectively (Additional file 1). A total of 12,680, 13,310, 12,650, 12,595 genes were found expressed (average CPM ≥ 1 considering all 100 samples) in liver, lung, spleen, and muscle, respectively. Of these, less than a fifth presented high expression values (CPM ≥ 100) (Additional file 2). PCA analysis showed clear clustering based on tissue type, with lung and spleen being the closest, as expected due to their similar immunity response [41] (Additional Fig. 3). Tissue-specific genes, that showed 4 times higher expression in a specific tissue compared to the other tissues, were identified: 1,107, 668, 362, and 952 in liver, lung, spleen, and muscle, respectively. These genes presented clear contributions to the main functions of their tissue as found in a gene ontology analyses (Additional file 3).

### eGWAS analysis

After quality control, 535,896 SNPs and all 100 samples remained for the association analysis (Additional file 4). PCA clustering of the crossbred study samples and purebred parental lines reflects the 3-way cross as expected (Additional Fig. 4).

Across the four tissues, the eGWAS identified over 1 million significant associations, ranging from 200,076 for muscle to 340,540 for spleen (Table 1). Significantly associated polymorphisms were merged into eQTL regions based on vicinity (see Methods section) resulting in 94.4, 92.1, 98.0, and 85.3% of these associations could be merged into eQTL regions in liver, lung, spleen, and muscle, respectively. The remaining associations were not considered, as they could potentially be spurious associations with only one or two significant variants.

**Table 1:** The number of associations, eQTL regions, and genes per tissue obtained in the eGWAS analysis

Tissue	Number of associations	Number of eQTLs	Number of associated genes	
			Total	Protein coding (%)
Liver	222,858	4,293	1,003	913 (91%)
Lung	322,386	10,630	2,405	2,284 (95%)
Spleen	340,540	4,533	1,258	1,156 (91%)
Muscle	200,076	6,871	969	881 (90.9%)

For each tissue, over 4,000 eQTL regions were found with lung (10,630) and liver (4,293) as the highest and lowest, respectively (Table 1; Additional file 5). On average, 1,400 genes were found associated with eQTL regions, ranging from 969 in muscle to 2,405 in lung (Table 1). Most of the genes (> 90%) were annotated as protein coding (Table 1; Additional Fig. 5). Almost 50% of the eQTL-associated genes were found associated in two or more tissue types but only 85 of these eQTL-associated genes were found simultaneously in all four tissues (Additional Fig. 6). The proportion of genes found associated to eQTLs did not differ notably across the different expression bins (e.g. low, moderate or highly expressed; Additional file 6). Also, no difference was observed when comparing *cis*- and *trans-* classified eQTLs (Additional file 6).

The observed eQTL regions varied in lengths with the majority (72%) being less than 5 Mb (Additional file 7; Additional Fig. 7). The average size of the eQTLs was 3.2, 2.8, 3.9, and 3.6 Mb in liver, lung, spleen, and muscle, respectively. Regardless of their length, polymorphisms within randomly selected eQTLs showed moderate (R^2^: 0.2) to high (R^2^: 1.0) LD between them, suggesting appropriate distance between two significant SNPs (see Methods) (Additional file 8).

### Annotation of *cis*- and *trans*-eQTLs

On average, 10% of the most significant variants from the eQTL regions were annotated in *cis*, 19% as *trans*-I and 71% as *trans*-eQTL-II (Table 2) (see Methods for definitions of *cis*- and *trans*-eQTL). Nevertheless, the proportion of these annotated eQTLs varied widely across tissues. The lowest number of *cis*-eQTLs was found in muscle (5%) and lung (6%), followed by liver (12%), and the highest proportion in spleen (18%). Also, spleen presented the largest proportion (31%) as *trans*-eQTL-I compared to the other tissues which were more predominantly classified as *trans*-eQTL-II (Table 2).

**Table 2:** Classification of *cis-* and *trans-*eQTLs (I and II) per tissue type

Tissue	Number of cis-eQTL (%)	Number of trans-eQTL (%)	
		I	II
Liver	495 (12%)	872 (20%)	2,926 (68%)
Lung	595 (6%)	1,371 (13%)	8,664 (81%)
Spleen	800 (18%)	1,395 (31%)	2,338 (51%)
Muscle	346 (5%)	815 (12%)	5,710 (83%)

**Fig. 1:**
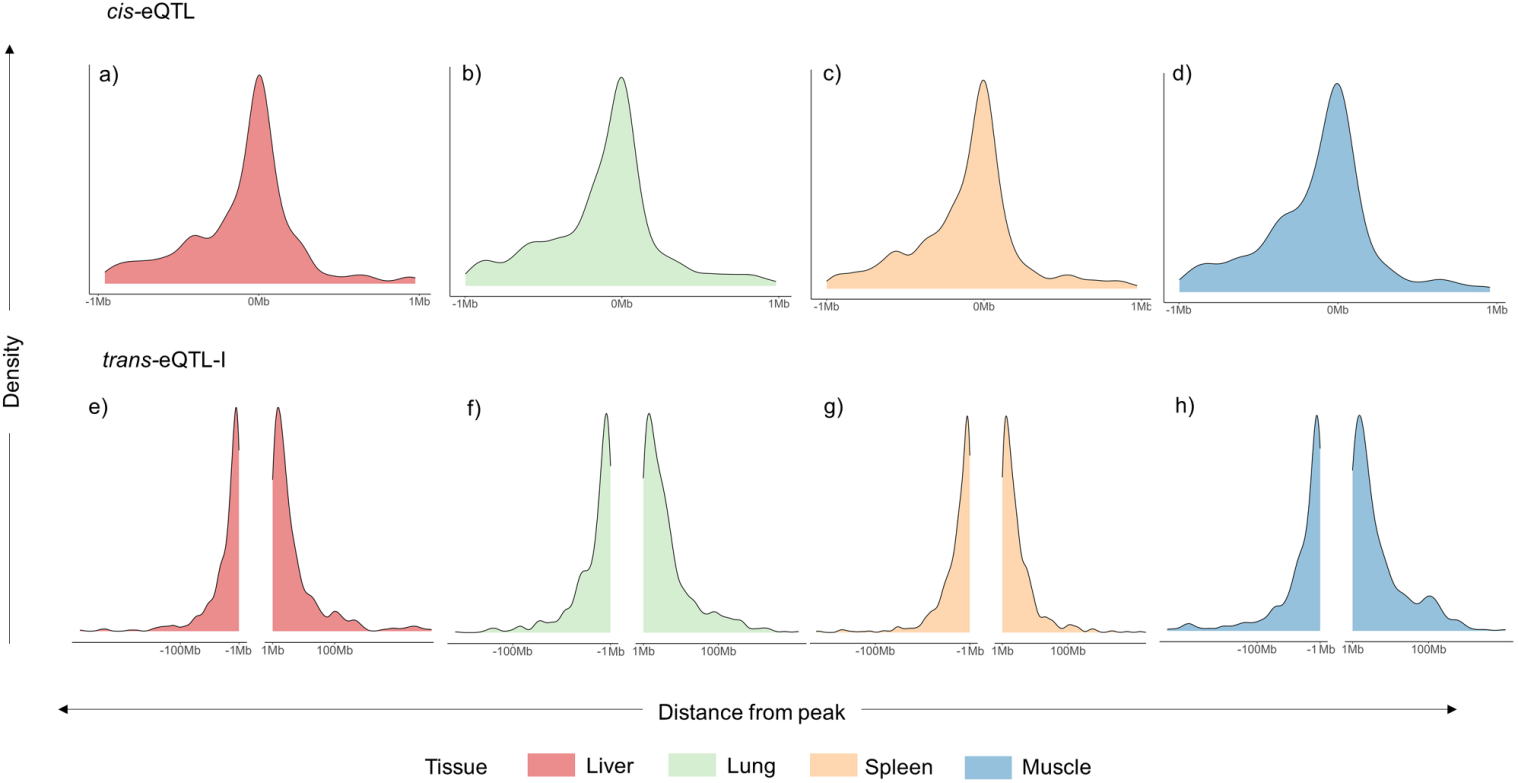
Density plot of the distance between the position of the most significant SNP and its associated gene. Density plots divided by *cis*-eQTL (a-d) and *trans*-eQTL-I (e-h) across the four tissues (see materials and methods for definition of eQTLs)

The distance between the most significant variant in the eQTL and its associated gene was evaluated for each tissue (Fig. 1). For *cis*-eQTL, the average distance was similar across tissues, around 2.5 Kb. For *trans*-eQTL-I, the average was between 20 (liver) and 34 Mb (muscle). Yet, median values indicate that for half of the associations the distances were below 18 Mb for all tissues.

**Fig. 2:**
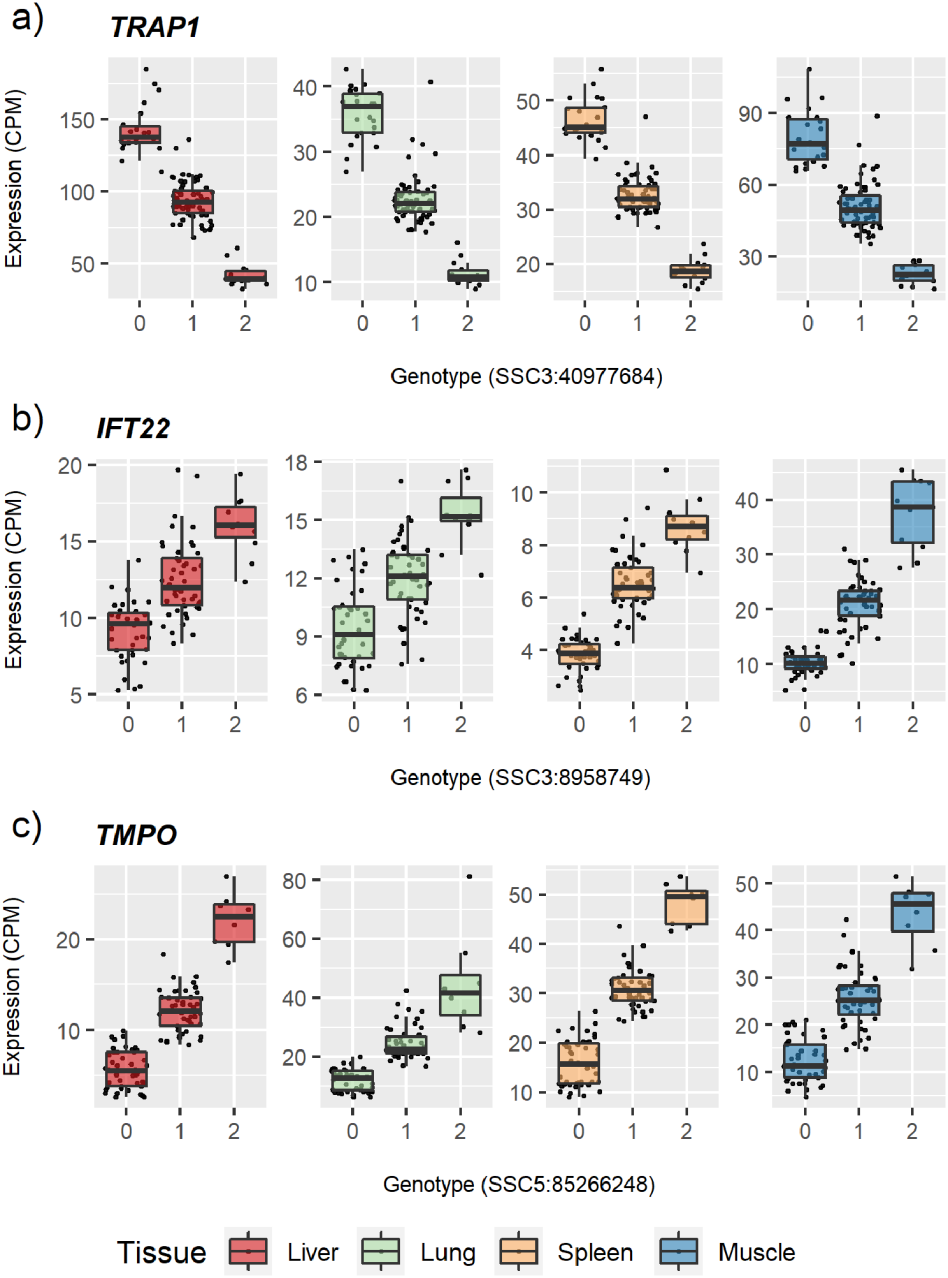
Boxplots with the association of genotypes and gene expression for genes identified. The difference in genotype (0 homologous for the reference alle; 1 heterozygous; 2 homologous for the alternative allele) is mediating gene expression (as CPM) across all the four tissues for the genes *TRAP1* (a), *IFT22* (b) *TMPO* (c). Each dot represents one sample. CPM: Counts Per Million

To identify the potential biological functions of the eQTL associated genes, gene ontology and pathway analyses were conducted. In these analyses, spleen was found significantly enriched for catabolic processes, while liver, lung, and muscle exhibited enrichment for genes involved in defense response to pathogens and (innate) immune response, among others. (Additional file 10). All four tissues were found enriched for metabolic pathways as well as other immune-related pathways as cytokine receptor functions (in liver), autoimmune roles (liver and muscle) or oxidative phosphorylation (in lung) (Additional file 11). Zooming in to genes with high significant association acting as *cis*-eQTL in all four tissues, we identified *TRAP1*, *IFT22* and *TMPO* with roles related to meat quality [42] and immunity [43] (Fig. 2).

For each eQTL, the association value of the eGWAS (*q*-value) and its annotated group distance (*cis* or *trans)* was assessed. The closest associations, classified as *cis*-eQTL, presented significantly lower *q*-values than those classified as *trans*-eQTL-I and *trans*-eQTL-II (Additional file 9; Additional Fig. 8). Moreover, significant differences between *trans*-eQTL-I and *trans*-eQTL-II were also detected in liver, lung, and spleen (Additional Fig. 8).

### eQTL map and *trans*-eQTL hotspots

**Fig. 3:**
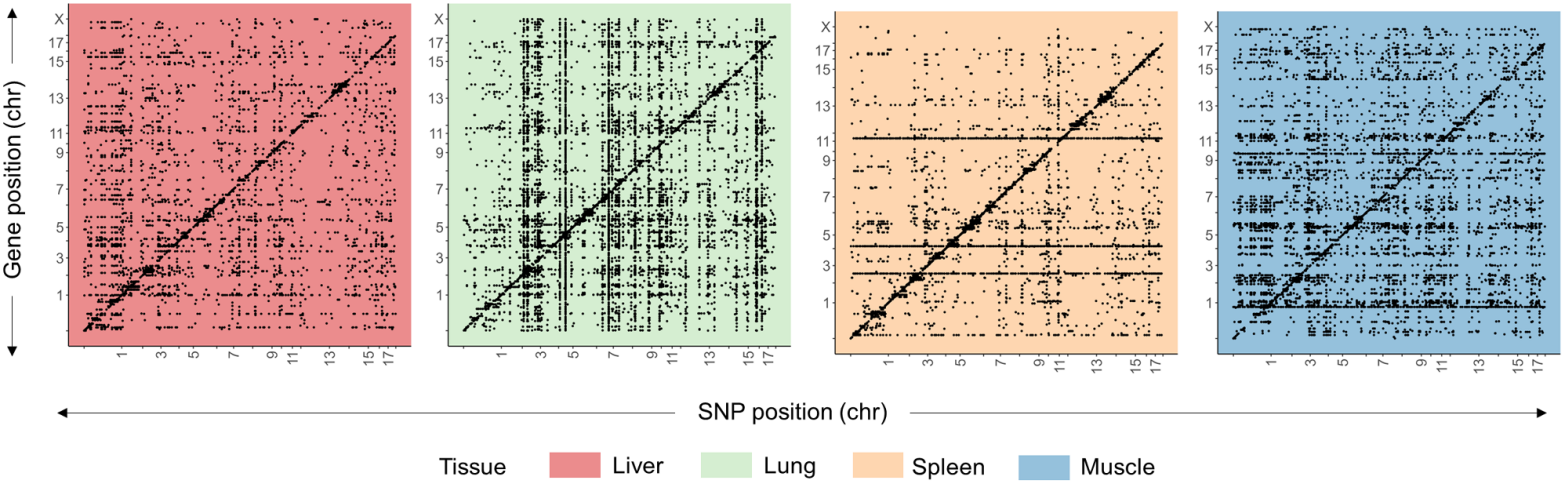
eQTL map across the four tissues. The genomic position of associated genes (y-axis) versus genomic position of the top polymorphism from the eQTL (x-axis). Each black dot represents a significant association between an associated gene and the most significant SNP of the eQTL region. Diagonal dots represent *cis-*eQTLs and off-diagonal dots represent *trans*-eQTLs. The presence of a vertical band suggests that numerous expressed associated genes are linked to a single genomic locus, indicating *trans*-eQTL hotspots. Conversely, a horizontal band indicate the association between many top polymorphisms from eQTLs and a single gene

The relationship of regulatory variation across the genome can be visualized with an eQTL map depicting each significant eQTL association as a dot (Fig. 3). The dots located on a diagonal line represent *cis*-eQTLs. A horizontal line indicates the association between many top eQTL polymorphisms and a single gene, most of them annotated as *trans*-eQTL. And last, multiple dots displayed as a vertical line suggests several genes associated with a single genomic locus, potentially an eQTL hotspot that could point towards a shared regulatory region or pathway (e.g. TF). Overall, the eQTL map, revealed different profiles for each tissue studied (Fig. 3).

**Fig. 4:**
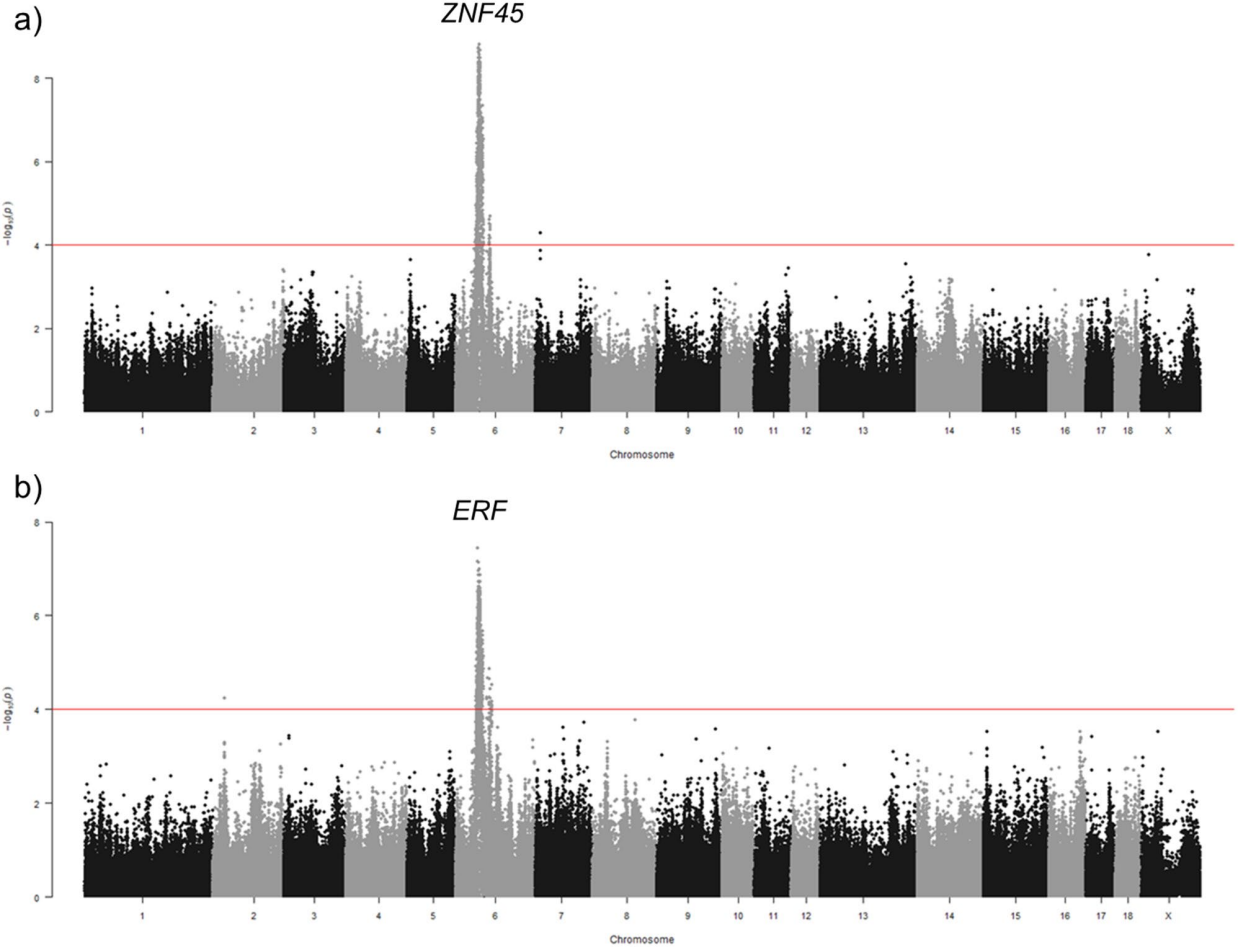
Manhattan plot of eGWAS for (a) *ZNF45* and (b) *ERF* in spleen. The x-axis represents chromosome positions (Mb), and the y-axis displays the - log10(P-value) of the genetic association. The horizontal red line is added to represent the genome-wide significance level (FDR ≤ 0.05)

Depending on the tissue, 13 to 22% of the eQTLs were found significantly associated with more than one gene (Table 3). A total of 20 (spleen) to 115 (lung) eQTL regions were associated with ten or more genes, defined as eQTL hotspots (Table 3; seen as a vertical line in Fig. 3). Each eQTL hotspot region was associated with up to 54, 56, and 82 genes for liver, spleen, and muscle, respectively. However, in lung, an eQTL hotspot was identified affecting a total of 639 genes. Among all of the eQTL hotspot regions (Table 3), most of the associated genes were annotated as *trans*-eQTL and only a few presented genes acting as *cis*-eQTL: 1 eQTL region with 1 gene (in liver), 5 eQTL with 19 genes (in lung), 1 eQTL region with 4 genes (in spleen), and 2 eQTL regions with 2 genes (in muscle) (Additional file 12). Remarkably, only two of these genes acting as *cis*-eQTL in spleen were annotated also as TFs (Additional file 12). They were *ERF* and *ZNF45* (Fig. 4), from the TF family ETS and zf-CH2, respectively. The region on SSC5:50,380,082:54,714,416 in lung affecting 639 genes did not show any candidate TF gene acting as *cis*-eQTL (Additional file 12).

**Table 3:** Number of eQTLs associated with a single gene or multiple genes

Tissue	Number of eQTLs associated with 1 gene	Number of eQTLs associated with > 1 gene	Number of eQTLs associated with ≥ 10 genes
Liver	2,273	340	46
Lung	2,351	484	115
Spleen	3,024	443	20
Muscle	2,425	685	111

Number of eQTLs associated with ≥ 10 genes are thus also included also in the second column (> 1 gene)

The role of the genes within eQTL hotspot regions was further assessed. Overall eQTL hotspot genes were enriched for regulation of immune response and response to viruses, in all four tissues, as well as for the mitochondrial respiratory chain complex (in lung), among others (Additional file 13). Secondly, potentially shared TFs binding sites were queried through motif analysis of the promoter sequence for each of the genes within the eQTL hotspot. For this, 7 eQTL hotspots shared across the four tissues were used (Additional file 14). Between 2 and 4 known motifs were found for each eQTL hotspot (Additional file 14). A total of 56 TFs were detected in the vertebrate dataset of which 38 were also annotated in pig [3] (Additional file 14). Nevertheless, none of these matched TFs was found close to its eQTL hotspot region.

## Discussion

Investigating the genomic regions and molecular processes associated with porcine health and robustness has become a major focus of interest due to its increasing relevance for the sustainability of pig breeding and production [44]. Remarkably, most efforts to date have focused on a single tissue type and purebred lines. Yet, studying different tissues can provide a comprehensive understanding of complex phenotypes. Moreover, commercial female pigs are mostly a cross of two purebred sow lines whereby crossbred animals can benefit from heterosis [45], and even be used in genetic evaluations to maximize the genetic response of purebred lines [46–48]. The finisher pig for meat production is the offspring of the crossbred sows mated to a third line, a sire line, mainly selected for high growth and feed efficiency. Thus, in this study, an exhaustive eGWAS was performed in 100 samples in four tissues to identify regulatory variants in young prepubertal female animals of this three-way cross in four different tissues with key roles in body energy homeostasis and immunity [49–52].

### mRNA profiles across liver, lung, spleen, and skeletal muscle

The RNA-seq analysis resulted in over 12,000 genes expressed across the four tissues with clearly differentiated profiles (Additional Fig. 3), most likely as a result from their distinct embryonic origins, immune response, and physiological functions [41, 53, 54].

In liver, in agreement with previous studies [49, 55], its critical role was confirmed in producing enzymes and proteins necessary for the metabolism of sterol, alcohol, lipid, organic, and amino acid as well as other substances (Additional file 3). The most highly expressed genes included *ALB* which codes for albumin, the most abundant protein in liver [56], *APOE* (Apolipoprotein E) and *APOC3* (Apolipoprotein C3), both involved in cholesterol metabolism [57] and *COX1* (Cytochrome C oxidase subunit 1), involved in contributing to inflammatory responses [58].

In lung, porcine studies have mostly focused on differential expression after challenge with viruses [59]. In lung, we found several tissue specific genes including *DNAAF1*, mainly involved in the structure and function of cilia [60], which play a vital role in transporting mucus, pathogens and toxins out of the airways [61]. The highest expression in lung was found for the gene *SFTPC* (Surfactant Protein C), which has a role in maintaining the stability of pulmonary tissue in pigs [62].

Spleen, as a lymphoid organ, was predominantly enriched for immune system processes (Additional file 3), aligning with its role in innate and adaptive immunity [36, 53]. *IGHM* was the most highly expressed gene and encodes the C region that defines the immunoglobulin isotype. Immunoglobulins are responsible for identifying and neutralize invading pathogens, bacteria or viruses [63].

Skeletal muscle has been extensively studied using RNA-seq [18, 64–66]. Most expressed genes are involved in muscle function and maintenance, including muscle contraction, movement, development, differentiation, structure and organization (Additional file 3). Two of the highest expressed genes were *MYH2* (Myosin-2) and *ACTA1*. *MYH2* is involved in skeletal muscle contraction [67] and its lncRNA expression has been found differentially expressed in back fat tissue from different growth stages in pigs [68]. *ACTA1* (Actin Alpha 1, Skeletal Muscle), is a member of the actin family of proteins which are the major constituents of the assembly of muscle filaments, development of skeletal muscle fiber and cell motility [69].

### eGWAS analysis, cis and trans-eQTLs

The eGWAS resulted in over 1 Million significant associations across the four tissues and significant polymorphisms were annotated for over 26,000 eQTLs. Although our sample size can be considered small with high number of associations, it should be noted that out of the more than 6.7 B SNP-gene associations per tissue performed, less than 0.005% were found significant. The nature of our eGWAS results are in line with similar studies in porcine [10, 18]. The strength in our study is potentially amplified by the nature of the population used – cross breed animals that are likely present a higher degree of genetic diversity than in a purely bred population [70]. The eQTLs were associated with a total of 4,262 different genes of which only 2% were shared across the four tissues. In agreement with previous studies [18], common regulatory variants are less frequent than tissue-specific variants. Lung and spleen shared the biggest proportion of eQTL associated genes, as expected due to their shared role in the immune response [41]. Remarkably, lung presented the largest number of eQTL regions (10,630) and the majority were annotated as *trans*-eQTL-II (similar pattern as the other three tissues). Although no previous work has been done in porcine lung eGWAS, in human eGWAS, similar results with high number of eQTL regions have been reported [71], potentially indicating distal and complex genome regulation. Muscle presented the second largest number of eQTLs, with the biggest average *trans*-eQTL-I length (34 Mb) and the second largest for *cis*-eQTL (2.5 Kb). This could be related to the genetic difference between the sire and the sow lines. The terminal sire line has been selected for high growth, feed efficiency, and lean meat percentage, thereby influencing muscle growth and structure [72], whereas the sow lines show high fertility and mothering ability. In this three-way cross, especially variants fixed in the sire line will result in large eQTL regions due to high extent of LD.

Although the total number of *cis*-eQTLs was lower than *trans*-eQTLs (I and II) (Table 1), *cis*-eQTLs presented more significant polymorphisms per eQTL (average of 67 polymorphisms across tissues) compared to *trans*-eQTLs (15). Moreover, *cis*-eQTL regions exhibited significantly lower *q*-values between their most significant variant and its associated gene. This is in line with previous studies [10, 18, 73] and can be explained by the fact that the variant directly impacts the expression of the corresponding *cis*-eQTL associated gene or is in close linkage disequilibrium to the causal variant [74]. Moreover, *trans*-eQTL associated genes may involve an additional intermediate step, such as a regulatory gene, which could cause additional noise and lead to less significant differences explaining the higher *q*-values observed [74]. Another hypothesis is that these *trans*-eQTL could be distant enhancers playing a role in adjusting optimal expression. Therefore, variation in these regions is less likely to have a large impact.

Considering the genes within the top 10% *cis*-regulatory associations, 5 were acting across all four tissues and are involved in porcine meat production and immunity traits. This includes *HUS1*, recently reported in an eGWAS porcine study [18], which has been correlated with porcine intramuscular fat [75]. Similarly, *TRAP1* which plays a role in intracellular calcium concentration [76] has been reported as a candidate gene for pork meat pH [42] (Fig. 2). Other shared genes include *IFT22*, which contributes to intraflagellar transport, crucial for the assembly, maintenance, and function of cilia, cellular structures that serve as sensory and regulatory organelles [77] (Fig. 2) and *CCDC125*, which regulates cell motility particularly within the immune system and is predominantly expressed in lymphoid tissues (as spleen) [78]. The last shared gene was *TMPO*, which plays a critical role in regulating cell cycle progression, encoding a key component of the nuclear lamina, essential for maintaining nuclear stability and regulating gene expression [79] (Fig. 2).

To identify other interesting regulated genes per tissue, the focus was placed on *cis*-eQTLs that presented the highest ratio of significant variants after correcting for eQTL length. Genes identified included *BSCL2* in spleen, mutations in *BSCL2* have been associated with fat deposition in pigs [80], *TRAPPC9* in the liver, which has been implicated in porcine backfat thickness [81] as well as nutrient absorption and body size [82], and in in muscle, *CLCA2*, which is involved in mediating the calcium contraction response [83]. *CLCA2* has been earlier found in a haplotype region subject to strong opposite selection between Duroc (a sire line) and Large White (a sow line) pigs [84]. As well as the gene *TAOK1* in muscle, which in humans is related to muscle hypotonia and growth disorders [85], and in a previous GWAS study in pigs [86], it has been pointed as a candidate gene associated with the number of mummified Landrace animals.

### eQTL maps across tissues

The majority of eQTLs identified in this study were associated with a single gene (between 78 and 87% across the four tissues), but eQTLs associated with multiple genes are particularly interesting. These could point towards genomic loci affecting several genes, potentially involved in the same pathway, or highlight TFs or transcription co-factors (TcoFs) acting as regulators of gene expression. Among all of the eQTL hotspots identified, only 2 *cis*-eQTL in spleen were annotated as TF: *ERF* and *ZNF45*. The ERF (ETS2 Repressor Factor) protein belongs to the ETS TF-family and is present in several tissues in humans [87]. ERF has a strong transcriptional repressor activity and downregulates expression of genes involved in cellular proliferation [88]. ZNF45 is also a transcriptional repressor. It belongs to the family of Zinc-finger proteins (ZNFs) which is a diverse group of proteins that contain one or more zinc-finger domains, enabling them to interact with DNA, RNA, and other proteins. ZNFs have a variety of molecular functions and are among the most abundant groups of proteins [89].

We also attempted to find potential TF and TcoF shared across genes within eQTL hotspots by analysing DNA motifs in their promoter sequences. Although several TFs were identified, none were located close to their respective eQTL hotspot region. This lack of common regulatory regions could indicate that regulation of gene expression regulation occurs preferentially in a tissue-specific manner rather than a coordinated regulation across all tissues [19]; and/or that other regulatory elements may be playing a role in gene expression regulation, as long non-coding RNAs or micro RNAs [90, 91]; and/or that using whole genome sequencing data instead of genotypes can outperform the detection of TF and TcoFs [18].

Nevertheless, it is important to note that some genes may be under the influence of one or multiple other genes or genetic variants [92, 93]. Thus, even if not annotated as TFs, they might still play important roles in regulating specific pathways and modifying other genes’ expression [92]. This is the case for some *cis-*eQTL associated genes within eQTL hotspots. For example *ADSS2* in liver, encoding an enzyme that catalyses the initial step in AMP synthesis, has been significantly associated with average daily gain and loin muscle area traits in pigs [94]. Likewise, *FUT2* in lung, encoding an enzyme catalyzing the final step in the synthesis of the H antigen, has been linked to *E. coli* resistance in weaned pigs [95]. Two other examples are the *LIPE* gene in spleen, that in humans plays a critical role in the mobilization of cellular fat stores [96] and *SESN3* in muscle, a stress-sensitive gene that regulates lipid metabolism, directly controlling skeletal muscle fat metabolism [97] and found to play an important role in porcine skeletal muscle growth [97].

Our results point towards candidate regulatory variants of genes of interest for certain phenotypic traits. However, the role of many candidate variants on phenotypes remain to be elucidated. For this, future work includes breeding companies genotyping a subset of these SNP and study if there is an association between these genomic variants and phenotypes of interest, including immunity, metabolism, feed-efficiency, etc. In addition, overlapping eQTLs with QTLs can be particularly valuable in pinpointing functional mutations responsible for phenotypic variation. This overlap can help identify candidate genes and variants that are functionally relevant, thereby enhancing our understanding of the genetic basis of complex traits. Moreover, our study will also provide additional resources for the PigGTEx community [19], helping to close knowledge gaps between the expression of genes for each tissue and external factors, as age, sex, environment, genetic background or experimental design.

## Conclusions

Despite the unprecedented progress in identifying genetic loci that play a role in porcine traits, there is a lack of mechanistic understanding of how porcine traits like immunity or robustness are regulated in pigs. Here, we provide a unique dataset to investigate regulatory regions using 100 animals in four different tissues. Interestingly, the largest variability in regions affecting gene expression was found in the lung with more than 10,000 eQTLs. This newly cataloged repertoire of regulatory regions in the pig genome effective in young growing female finishers is now publicly available alongside with this manuscript in supplementary material. Our findings underscore the importance of tissue-specific regulation, interactions between loci and expression differences due to selection for different trait complexes in specialized sow and sire lines. These results provide a basis in understanding the complex genotype-phenotype interaction for further exploration in sustainable pig breeding and production.

### Electronic supplementary material

Below is the link to the electronic supplementary material.

Supplementary Material 1

Supplementary Material 2

Supplementary Material 3

Supplementary Material 4

Supplementary Material 5

Supplementary Material 6

Supplementary Material 7

Supplementary Material 8

Supplementary Material 9

Supplementary Material 10

Supplementary Material 11

Supplementary Material 12

Supplementary Material 13

Supplementary Material 14

Supplementary Material 15

Supplementary Material 16

Supplementary Material 17

Supplementary Material 18

Supplementary Material 19

Supplementary Material 20

Supplementary Material 21

Supplementary Material 22

Supplementary Material 23

## Data Availability

The datasets RNA-seq generated and/or analysed during the current study are available at NCBI’s BioProject PRJEB67310. Bioinformatic scripts can be found at: https://gitlab.com/samin.h.farhangi as pps-lift-1 and pps-lift-2.

## Abbreviations

ANOVA: One-way analysis of variation
CPM: Count per million
eGWAS: Expression GWAS
FarmGTEx: Farm animal genotype-tissue expression
GO: Gene ontology
GWAS: Genome-wide association studies
LD: Linkage disequilibrium
LSD: Least significant difference
PCA: Principal component analysis
RIN: RNA integrity number
SNPs: Single nucleotide polymorphisms
TF: Transcription factor
TcoF: Transcription cofactor
