# Supplementary material for: Expression genome-wide association study identifies key regulatory variants enriched with metabolic and immune functions in four porcine tissues
Source: BMC Genomics. 2024 Jul 11;25:684. doi: 10.1186/s12864-024-10583-w (PMC11238464; doi:10.1186/s12864-024-10583-w)
Supplement: Supplementary file 1 — Supplementary Material 1 [file 12864_2024_10583_MOESM1_ESM.docx]

**Additional files:**

**Additional figure 1** (TIFF)

Overview of the RNA-seq analysis workflow including the software, versions, and parameters.

**Additional figure 2** (TIFF)

Principal Component Analysis (PCA) of the 100 RNA-seq samples. Overall sample variability and batch effect was assessed the across the four tissues.

**Additional figure 3** (PNG)

Principal Component Analysis (PCA) based on expression levels of all genes for the four tissue types studied. Each dot represents a sample.

**Additional figure 4** (TIFF)

Principal Component Analysis (PCA) analysis based on genotypes of the purebred lines and the three-way crossbred samples used in this study. Breed lines included were: Large White (dark blue), Landrace (gold), Synthetic boar (brown), and the crossbreed samples (Landrace*LargeWhite)*Synthetic (light blue).

**Additional figure 5** (TIFF)

The proportion of associated genes identified from the eGWAS analysis across the four tissues as protein coding, long non-coding RNAs or other types of gene biotypes. a) liver, b) lung, c) spleen and d) muscle, categorized by its annotation as: protein coding, long non-coding RNA (lncRNA) or others (pseudogene, Mt-rRNA, Mt-tRNA, miRNA, IG_V_gene, rRNA, snoRNA, Ribozyme).

**Additional figure 6** (TIFF)

Venn diagram displaying the overlap of associated genes identified in the eQTL analysis across tissues.

**Additional figure 7** (TIFF)

Density plot of the length of eQTL across the tissues. Density plot for a) liver, b) lung, c) spleen, and d) muscle. The x-axis indicates the eQTL length and the y-axis the density of eQTL within each length.

**Additional figure 8** (TIFF)

Boxplots with significant associations (as *q*-values) obtained in the eGWAS analysis. Associations are divided in *cis*-eQTL, *trans*-eQTL-I and *trans*-eQTL-II and across the four tissues. Columns with different letters were found to be statistically different with Fisher’s Least Significant Difference (LSD), P-value < 0.05.

**Additional file 1** (XLS)

Sample information and RNA-seq results. Family ID refers to mother ID. Batch numbers indicates the slaughter batch day. Number of reads that passed the quality control as well as the percentage of mapped reads per each sample and tissue type.

**Additional file 2** (XLS)

Number of genes detected based on average expression (normalized as CPM) across the 100 RNA-seq samples. Divided by tissue type: liver, lung, spleen and muscle and different expression bins.

**Additional file 3** (XLS)

Biological process terms identified based on gene ontology enrichment analysis of tissue specific expressed genes in each tissue. Tissue-specific genes were considered if they had an average expression x 4 times larger than the other tissues.

**Additional file 4** (XLS)

Results of genotyped polymorphisms before and after filtering for quality parameters with PLINK. Results are provided per chromosome and total.

**Additional file 5** (XLS)

Significant eQTL regions annotated across the four tissues. For each eQTL, information is provided on its length (chromosome, start and end position), SNP ID of the most significant polymorphism and its position as well as the total number of significant polymorphisms found in the given eQTL. The table also includes information of the associated gene: Ensembl ID, gene symbol, strand and biotype. Information on the distance between most significant SNP and associated gene is provided (*cis* or *trans*), and the distance (in bp) between the most significant SNP and the gene’s start.

**Additional file 6** (XLS)

Proportion of expressed genes that were found as associated genes in the eQTL analysis. The table shows the proportion of genes across the different bins of expression in counts per million (CPM) separated between the *cis* and *trans* associated genes across the four tissues: liver, spleen, lung, and muscle.

**Additional file 7** (XLS)

Heatmap with the number of eQTL annotated across increasing intervals length. Distribution split by tissue type.

**Additional file 8** (XLS)

Linkage disequilibrium between significant polymorphisms within eQTL regions. The calculation is based on a subset of random eQTLs for the different tissues. Linkage disequilibrium, measured as R^2^, varied between moderate (0.2) to high (1.0).

**Additional file 9** (XLS)

Results of ANOVA test to investigate the significant variation of *q*-values among the three eQTL categories: *cis-*eQTL, *trans*-eQTL-I, and *trans*-eQTL-II.

**Additional file 10** (XLS)

Biological process terms identified based on gene ontology enrichment analysis of associated genes per each tissue.

**Additional file 11** (XLS)

KEGG terms identified based on KEGG pathways analysis of associated genes per each tissue.

**Additional file 12** (XLS)

Annotated eQTL which are associated with 10 or more genes. For each gene, information is provided on whether it is annotated as TF or TcoF, and its location as *cis* or *trans*.

**Additional file 13** (XLS)

The biological process terms identified on GO enrichment analysis of genes associated with eQTL hotspots.

**Additional file 14** (XLS)

Identification of motif structures in shared eQTL regions across all tissues. Analyzed using MEME and TOMTOM, and recognized as TFs in the JASPAR dataset.
