# Supplementary figures and images for: Expression genome-wide association study identifies key regulatory variants enriched with metabolic and immune functions in four porcine tissues
Source: BMC Genomics. 2024 Jul 11;25:684. doi: 10.1186/s12864-024-10583-w (PMC11238464; doi:10.1186/s12864-024-10583-w)

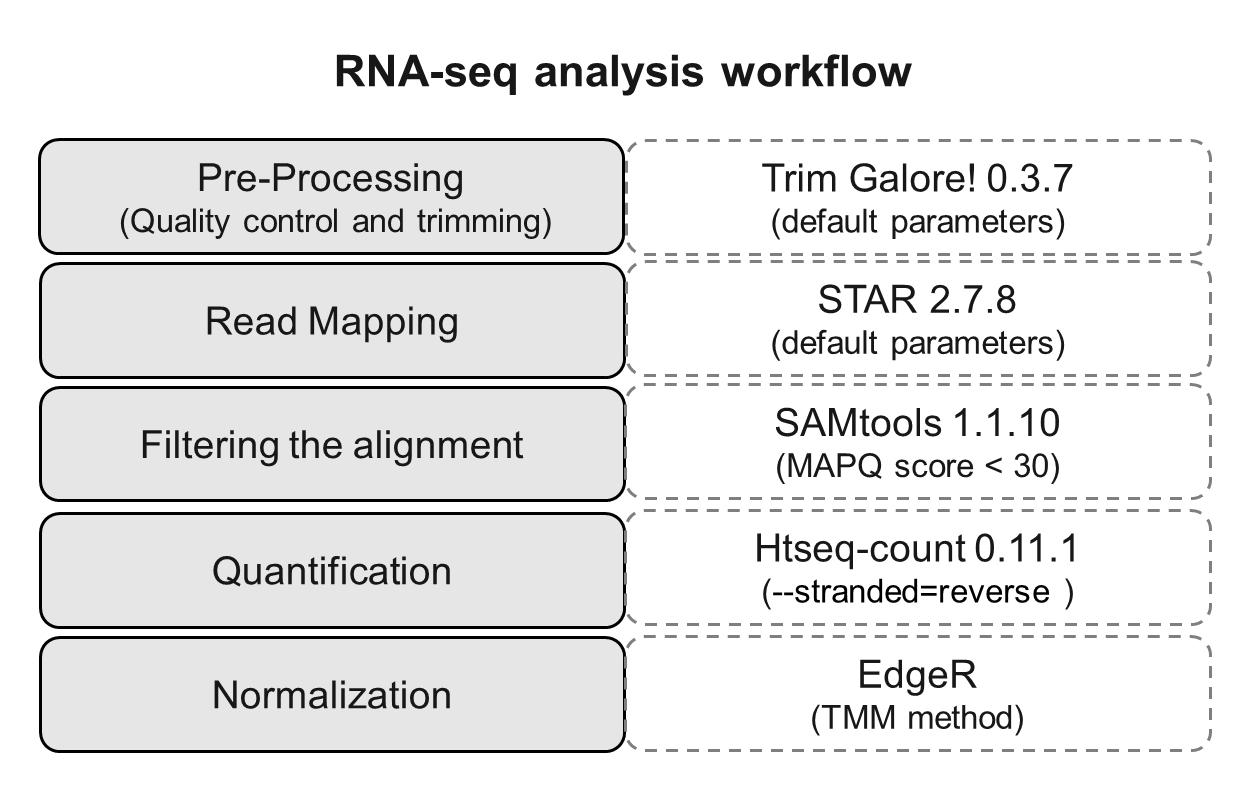

Supplement: Supplementary file 2 — Supplementary Material 2 [file 12864_2024_10583_MOESM2_ESM.tif]

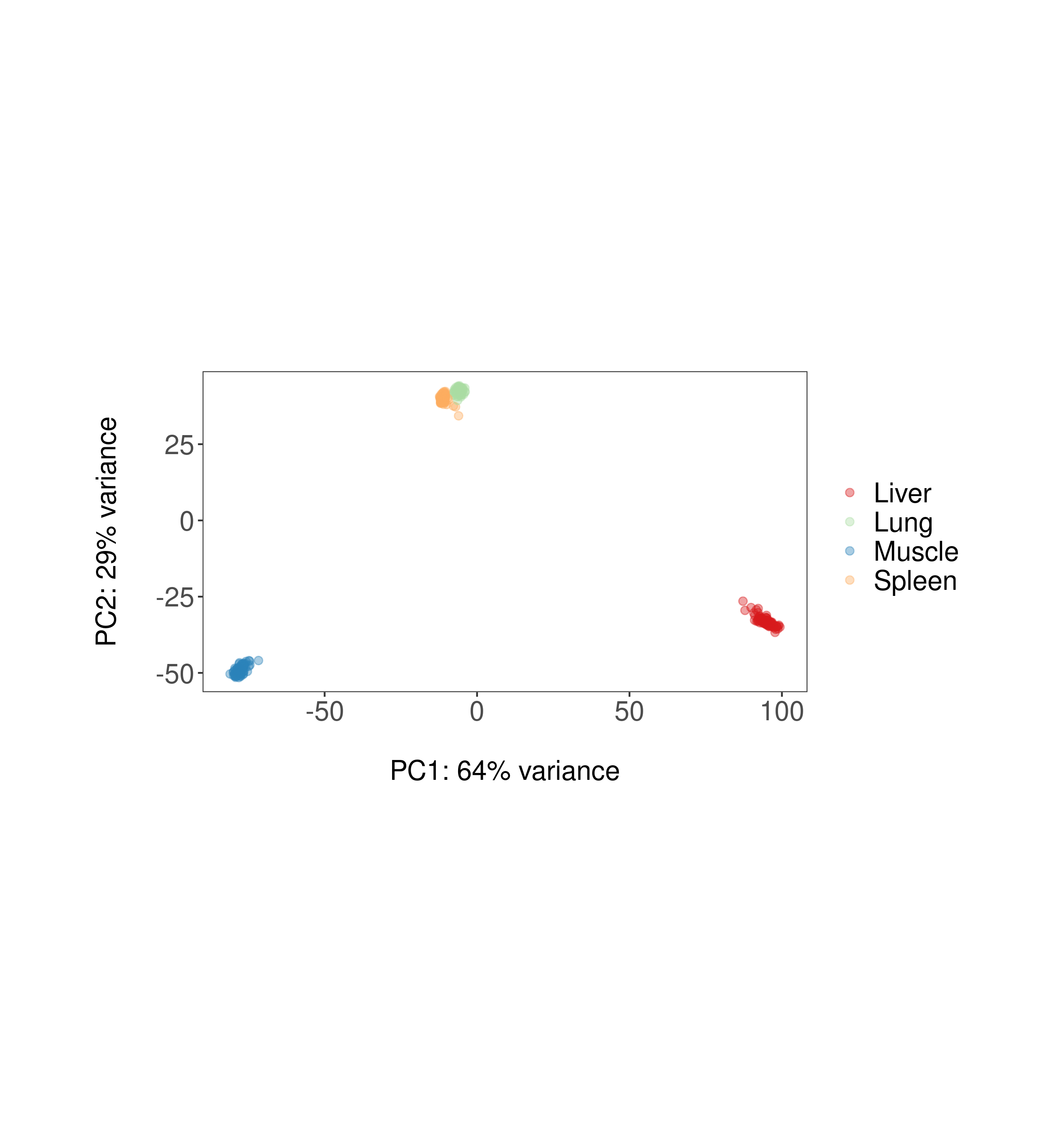

Supplement: Supplementary file 3 — Supplementary Material 3 [file 12864_2024_10583_MOESM3_ESM.png]

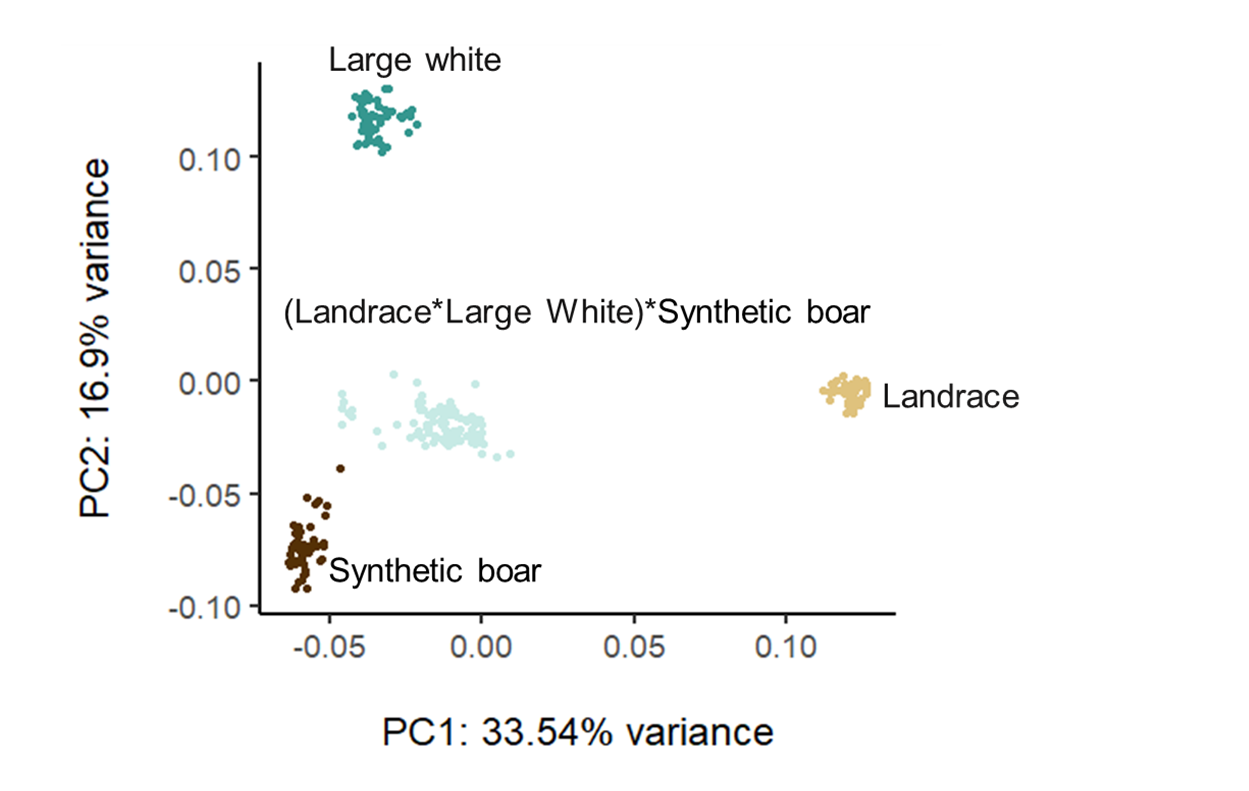

Supplement: Supplementary file 4 — Supplementary Material 4 [file 12864_2024_10583_MOESM4_ESM.tif]

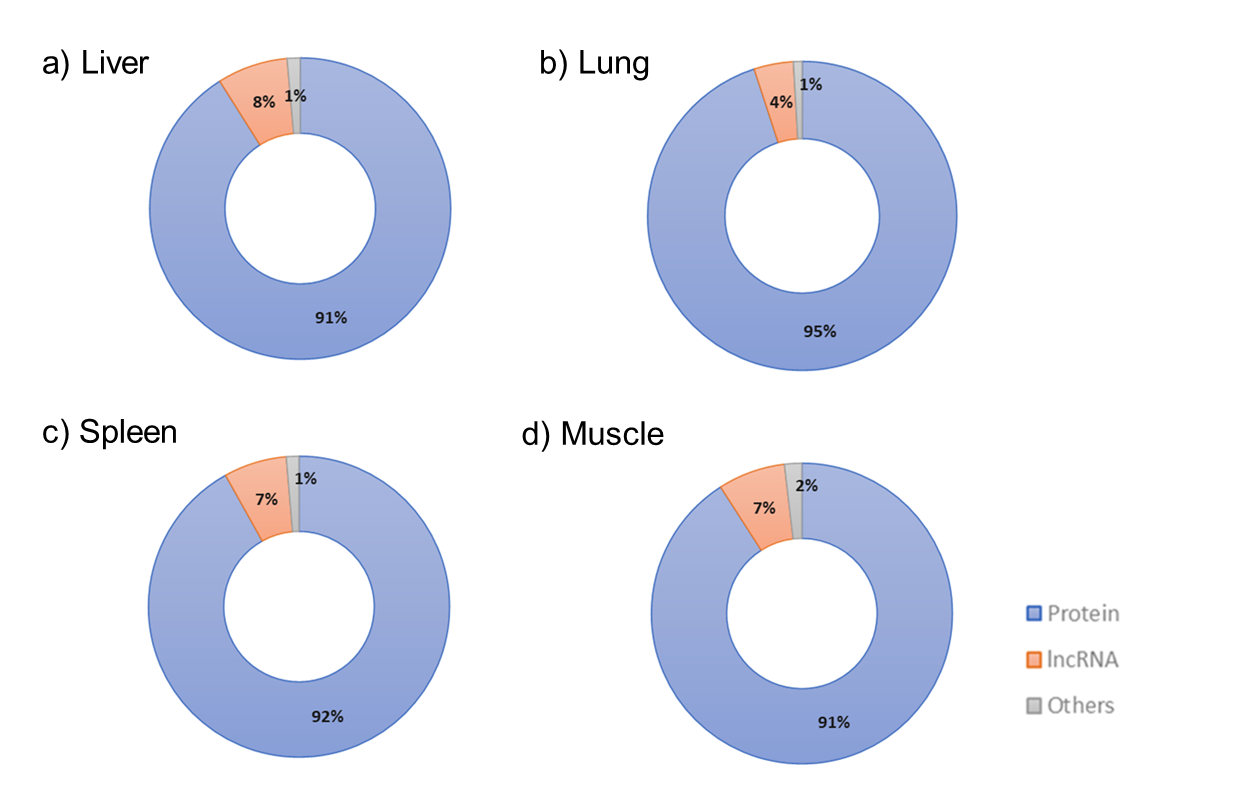

Supplement: Supplementary file 5 — Supplementary Material 5 [file 12864_2024_10583_MOESM5_ESM.tif]

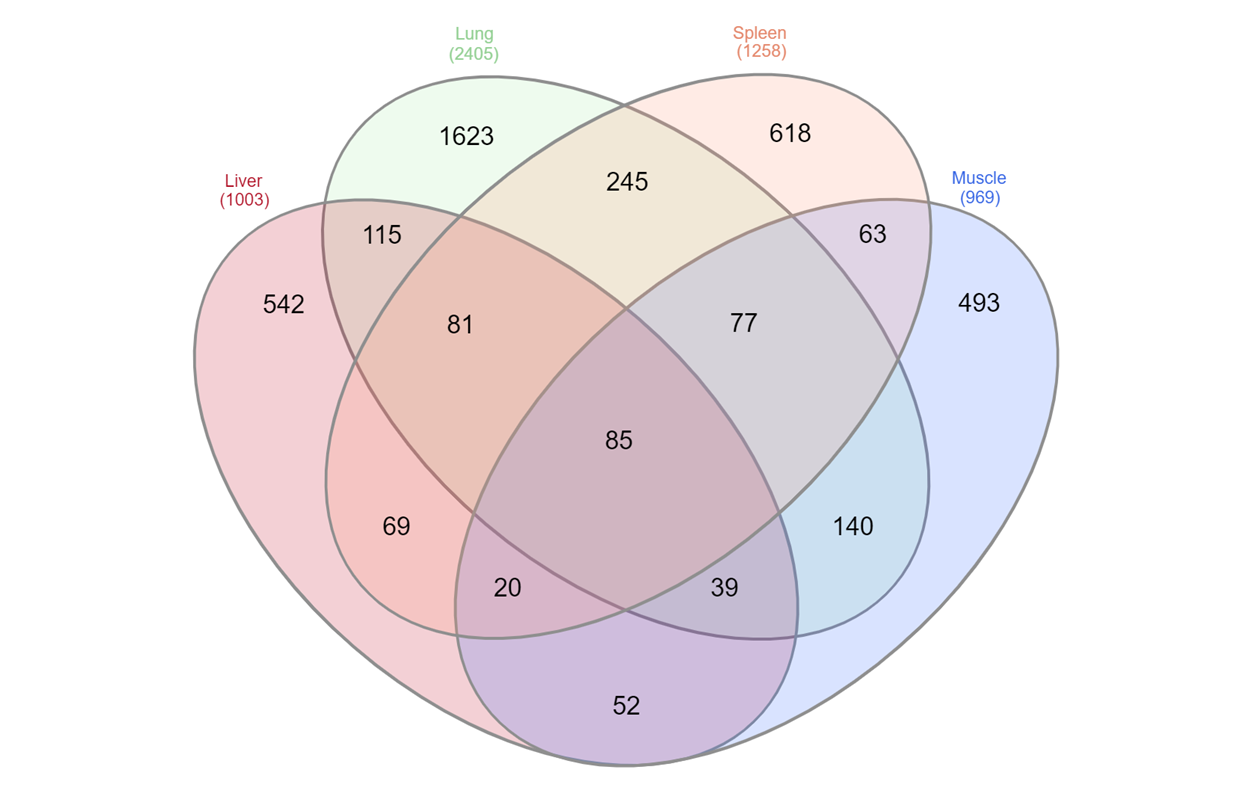

Supplement: Supplementary file 6 — Supplementary Material 6 [file 12864_2024_10583_MOESM6_ESM.tif]

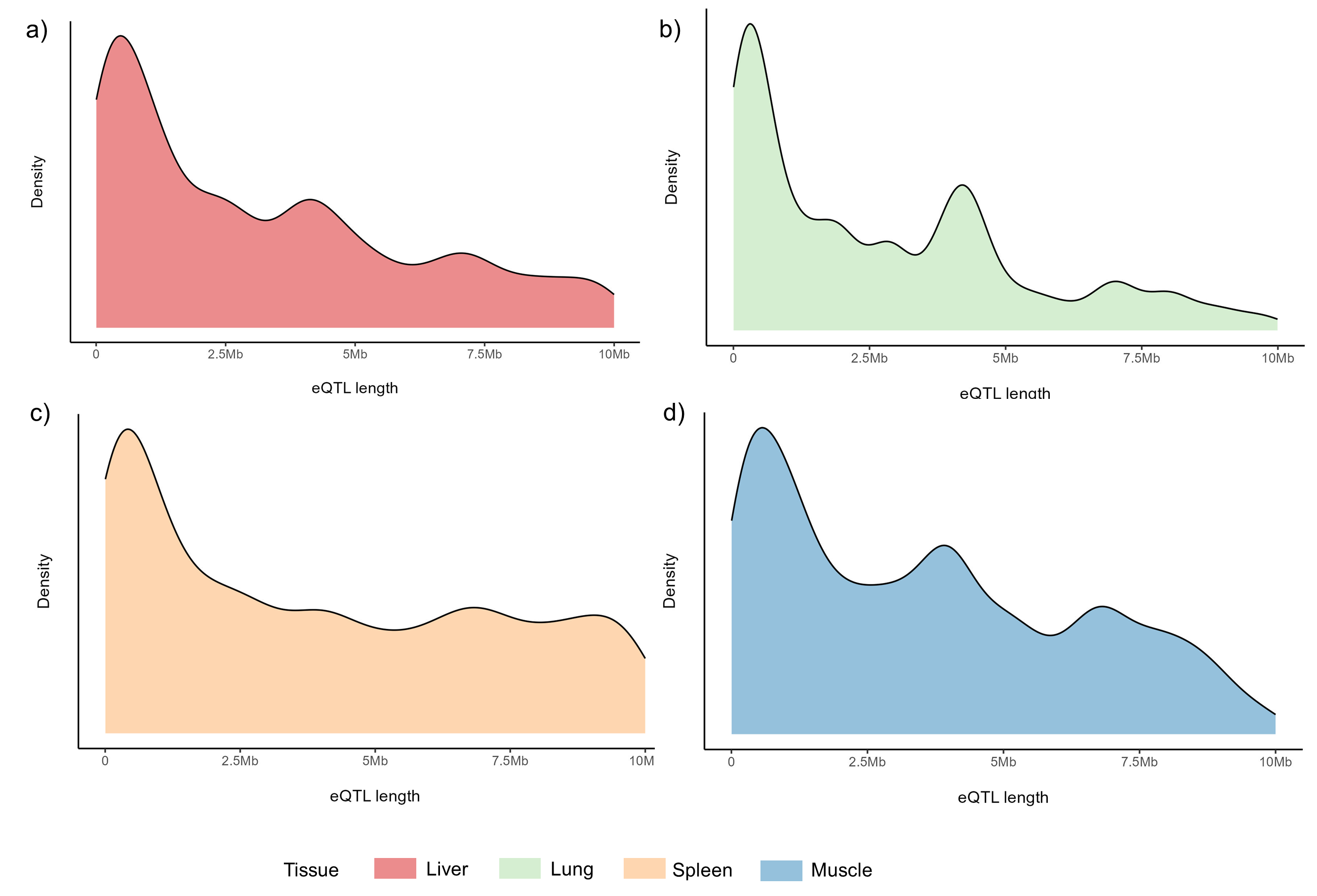

Supplement: Supplementary file 7 — Supplementary Material 7 [file 12864_2024_10583_MOESM7_ESM.tif]

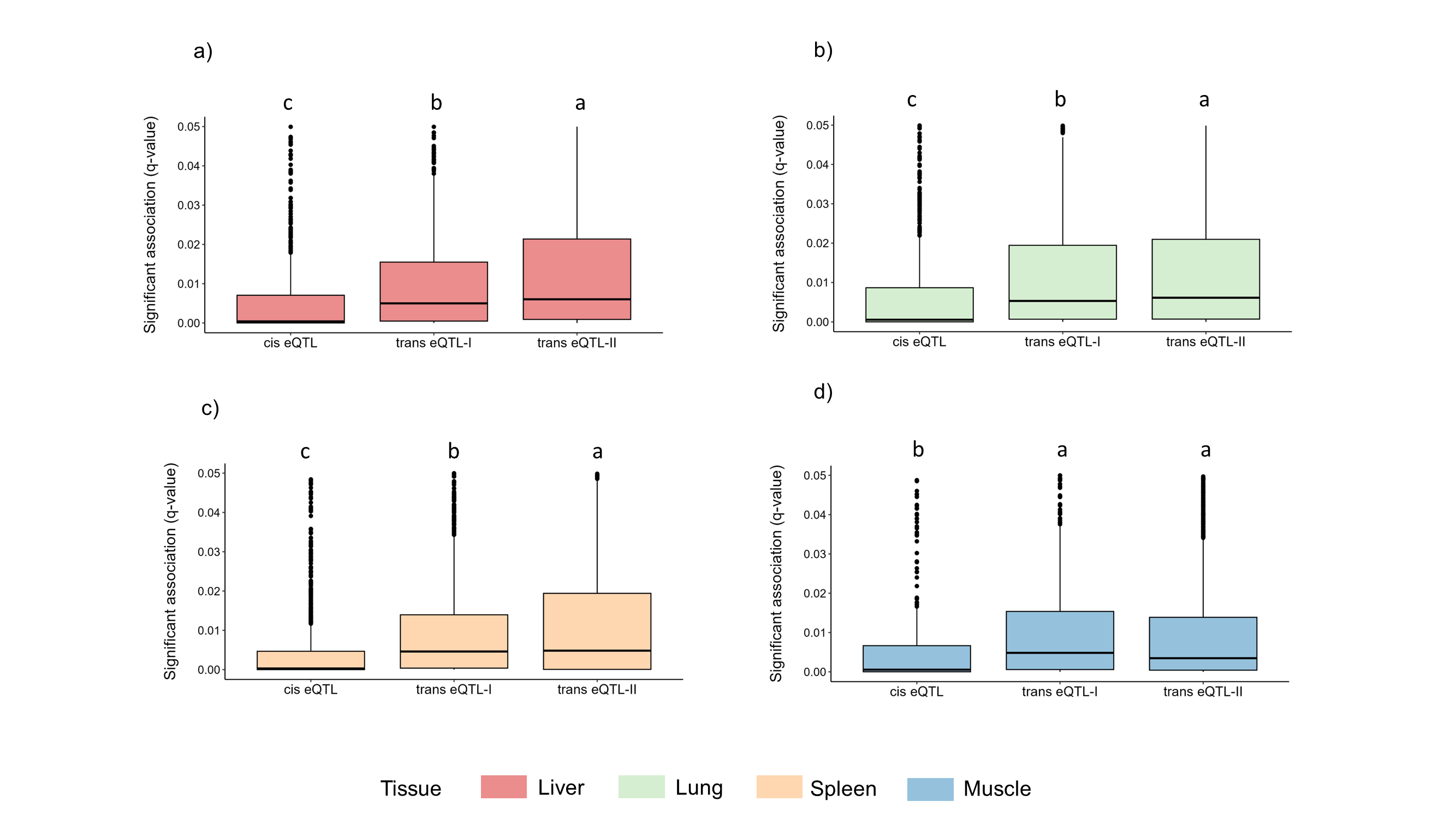

Supplement: Supplementary file 8 — Supplementary Material 8 [file 12864_2024_10583_MOESM8_ESM.tif]

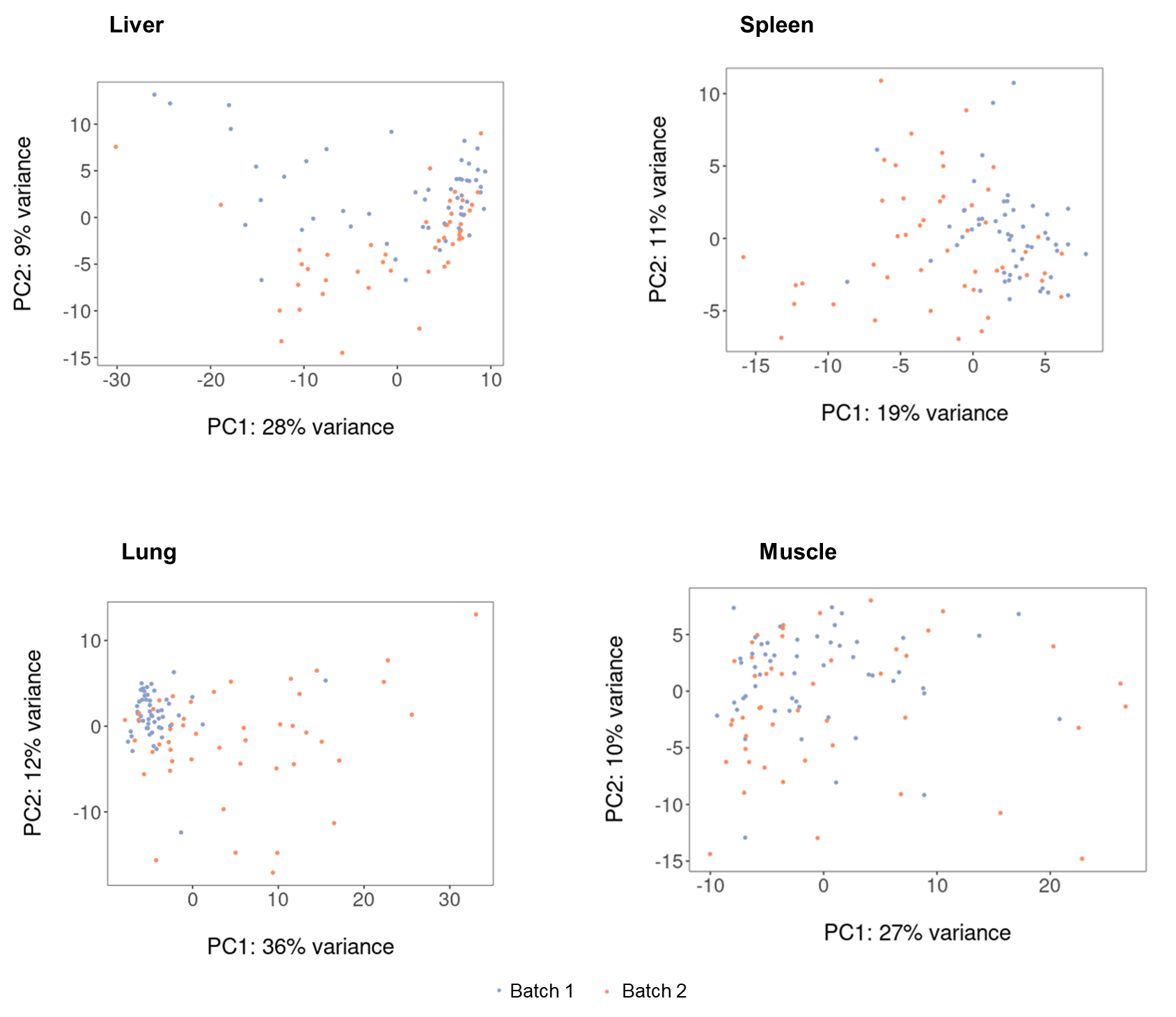

Supplement: Supplementary file 9 — Supplementary Material 9 [file 12864_2024_10583_MOESM9_ESM.tiff]
